# Single-cell RNA sequencing for the study of kidney disease

**DOI:** 10.1186/s10020-023-00693-8

**Published:** 2023-07-03

**Authors:** Jiayi Zhu, Jinrong Lu, Huachun Weng

**Affiliations:** The College of Medical Technology, Shanghai University of Medicine & Health Sciences, 279 Zhouzhu Highway, Pudong New Area, 201318 Shanghai, China

**Keywords:** Kidney disease, scRNA-seq, Pathogenesis

## Abstract

The kidney is an important organ for maintaining normal metabolism and stabilising the internal environment, in which, the heterogeneity of cell types has hindered the progress in understanding the mechanisms underlying kidney disease. In recent years the application of single-cell RNA sequencing (scRNA-seq) in nephrology has developed rapidly. In this review, we summarized the technical platform related to scRNA-seq and the role of this technology in investigating the onset and development of kidney diseases, starting from several common kidney diseases (mainly including lupus nephritis, renal cell carcinoma, diabetic nephropathy and acute kidney injury), and provide a reference for the application of scRNA-seq in the study of kidney disease diagnosis, treatment and prognosis.

**Supplementary Information:**

The online version contains supplementary material available at 10.1186/s10020-023-00693-8.

## Introduction

The kidney is a very important organ, which plays a vital role in maintaining the internal environmental stability and normal metabolism. The kidney has a complex structure with many different functions, which appears to be associated with different cell types. Some kidney diseases may possess cell type specificity, which to a certain extent increases our understanding of its pathogenesis (Liao et al. 2020; Wu and Humphreys 2017). Meanwhile, the heterogeneity of cell types in the kidney hinders the understanding of the pathogenesis of kidney diseases. Traditional RNA sequencing only evaluates the average gene expression of all different kinds of cells in the tissue. The rapid development of single-cell RNA sequencing (scRNA-seq) allows investigators to monitor global gene expression in individual cells, which can be of great help in identifying new cells, redefining known cells, and understanding cell differentiation.

Nowadays, new scRNA-seq studies related to kidney disease are emerging (Potter 2018). For example, Park et al. performed unbiased scRNA-seq of 57,979 kidney cells from healthy mice and successfully found that each cell type appeared to be important for the pathogenesis of kidney disease (Park et al. 2018). This has led to a deeper understanding of the application of scRNA-seq in exploring the pathogenesis of kidney disease.

### Common single-cell sequencing techniques

scRNA-seq is a rapidly evolving technology that can now be used to determine the precise gene expression patterns of tens of thousands of individual cells. This research tool allows us to classify homogeneous cells into different subtypes in greater detail, or to identify previously unknown cell types. Its basic steps include: single cell isolation, lysis of cells to obtain genetic material, whole gene amplification and sequencing analysis. Today, scRNA-seq is used in many fields such as microbiology, immunology, neurology and oncology, and has great potential for the diagnosis, treatment and prognosis of diseases (Wiedmeier et al. 2019). scRNA-seq is also an extremely effective method in kidney research. It allows us to observe various cell types and states at different stages. It can also be used to assess the reproducibility and reliability of kidney-like organs generated from pluripotent stem cells (Subramanian et al. 2019). In several studies in 2019, scRNA-seq successfully provided single-cell profiles of immune cells in the fetal and adult kidney and illustrated the differences between mature and immature kidneys, leading to a more comprehensive understanding of renal immune cells (Clark and Greka 2020). In summary, scRNA-seq has potential uses in the study of renal disease diagnosis, treatment and prognosis. It can provide a different way to study the pathogenesis of the kidney and can also add new potential options for diagnosis and treatment in the clinical setting. We summarized some of the most commonly used scRNA-seq platforms or methods, as shown in Table 1, providing a guide that could help nephrologists to choice scRNA-seq methods suitable for their studies.

**Table 1:** Principles and characteristics of different single cell sequencing

Methods	10X Genomics Chromium System	Fluidigm C1 System	Illumina /BioRad
Transcript coverage	5’ /3’ end-counting	Full-length	100 bp reads
unique molecular identifiers (UMI)	Yes	No	Yes
Single cell isolation	droplet-based microfluidic system	microfluidic-based system	Fluorescence-activated cell sorting
Cell size limitations	Independent of cell size	Homogenous size of 5–10,10–17,or 17–25 µM	Independent of cell size
Cell number required	≥ 20,000	≥ 10,000	Hundreds ~ thousands
Throughput	High	Low	High
Precision	High	Medium	High
Cost	Low	High	Medium
Operation	Easy	Difficult	Easy
Run time (approximately 5000 single cells)	~ 2–3 days	~ 3 weeks	~ 2 days

### 10X genomics

This is a droplet-based microfluidic system that can perform whole genome expression profiling on thousands of cells simultaneously. It works in a similar way to the usual droplet-based scRNA-seq method and consists of five steps: sample preparation, encapsulation in droplets, barcode incorporation and molecular amplification, sequencing, and data processing (Salomon et al. 2019). The main principle is a microfluidic “double cross” system where the cells are bound to Gel Beads at the first cross, and at the second cross they are encapsulated in oil droplets. The gel beads in the oil droplets contain specific DNA fragments including: Barcode, unique molecular identifiers (UMI) and Poly (T) which bind to the mRNA after cell lysis and form labelled cDNA by reverse transcription. Once the oil droplets burst, they are ready for amplification and the creation of cDNA libraries and sequencing.

InDrop and Drop-seq also use the same droplet-based microfluidic system and they use a similar principle of pairing individual cells with individual gel beads encapsulated in droplets, but there are some differences between these two and 10x Genomics. Firstly, 10x Genomics and inDrop use hydrogel beads, but Drop-seq uses brittle resin beads. In comparison, the hydrogel beads are larger, softer and more flexible, allowing a degree of avoidance of multiple cells or beads in a single oil droplet, and therefore they have a higher cell capture rate than Drop-seq. Secondly, 10x Genomics produces relatively less noisy data than inDrop, and it is simpler to operate and more capable of genetic detection. In addition, 10x Genomics has the advantage of low cost and high throughput, which allows it to be better used in complex experiments and to detect unknown cell populations.

However, 10x Genomics still has some problems. It cannot guarantee that each droplet contains a cell and a gel bead. Also, it cannot do full-length transcriptome sequencing. Compared to another scRNA-seq tool, Smart-seq2, it has a higher information loss rate for genes with lower expression levels and is less sensitive overall than full-length transcriptome sequencing because it can only transcribe from one segment (Salomon et al. 2019; Wang et al. 2021; See et al. 2018; Klein and Macosko 2017).

### The fluidigm C1 system

Fluidigm C1 is an automated microfluidic system that uses Integrated Fluidic Circuits (IFCs) and a highly automated microfluidic chip that can process up to 96 cells simultaneously. The roles of the IFCs includes cell capture, lysis, reverse transcription. When the system is filled with a cell suspension, individual cells are introduced into separate chambers while each captured cell can be viewed and confirmed under the microscope. After this, Fluidigm C1 performs the steps of cell lysis, reverse transcription, first-strand cDNA synthesis and amplification (Gong et al. 2018; DeLaughter 2018).

Fluidigm C1 offers high accuracy and sensitivity and generates full-length mRNA information with high quality gene expression readouts. However, as its counting and preparation requires a minimum of 10,000 cells, the platform is not suitable for the identification of rare populations in bulk cell samples. Moreover, one of its main limitations is that it is more stringent in terms of cell size and is only applicable to cells of relatively uniform size, resulting in some cells not matching the selected size of the chip being lost during capture and processing, so the characterisation of cell type composition in heterogeneous samples will be incomplete. In addition, the Fluidigm C1’s automated equipment and the microfluidic chip are expensive and not suitable for high volume sequencing (Zhu et al. 2020; See et al. 2018; Kolodziejczyk et al. 2015).

### Illumina bio-rad

The Human Genome Project is an important program in the field of the life sciences, paving the way for major technological developments in DNA and RNA sequencing. Solexa took the lead in developing next-generation sequencing (NGS, also known as massively parallel or high-throughput sequencing). After the company’s acquisition by Illumina, this technology was refined further and gave rise to a number of platforms that include the NextSeq, HiSeq and NovaSeq sequencers (Senabouth et al. 2020). In the Illumina® Bio-Rad scRNA-seq solution, Illumina combines NGS library preparation, sequencing, and analysis with Bio-Rad’s innovative droplet digital PCR (ddPCR) technology to provide a comprehensive, user-friendly workflow for scRNA-seq, supporting users for controlled experiments with multiple samples, processing conditions, and time points. This protocol enables transcriptome analysis of hundreds to thousands of single cells of multiple cell sizes in a single experiment (Khan et al. 1979). Among them, ddSEQ System is a NGS workflow for single-cell analysis, which can detect 8 samples simultaneously, and each sample can get 500 to 10,000 cells. Compared with other high-throughput capture platforms, Illumina® Bio-Rad ® capture only get mRNA information at the 3 ′ end, but the sequencing cost is relatively low. With the help of Bio-Rad’s Droplet Digital™ droplet separation technology, the platform can isolate and barcode single cells, complete single cell capture, amplification, expression profile library, and then downstream sequencing in the Illumina NextSeq500 instrument to obtain single cell expression profile data. The system can also use BaseSpace® Informatics Suite to perform primary and intermediate data analysis in Illumina’s cloud genomics computing environment, and can perform advanced data analysis and visualization using SeqGeq™ developed by LLC, FlowJo, a leading company in flow cytometry technology. While “second-generation” NGS technologies, such as Illumina technology, are also known as “short-read” sequencing technologies because the achievable read length is relatively limited, but large amounts of accurate sequencing data or “reads” can be generated through massively parallel settings (Winand et al. 2019). To date, most scRNA-seq methods require cDNA libraries to be compatible with short-read Illumina sequencing platforms. The Illumina platform is currently the most state-of-the-art platform, with reagents widely available, used, and benchmarked. As the most widely used and recognized platform in the field of metagenomics, the data sets generated by the Illumina platform (HiSeq 2000/4000) are used for the entire cross-platform comparison. The rapid update of its sequencing technology provides high-precision reads of up to 300 bp, and reads of 100 bp in length have been widely used by using the Illumina HiSeq technology (Natarajan et al. 2019; Chao et al. 2017).

### scRNA-seq in kidney disease research

The studies on renal diseases using scRNA-seq were summarized in Table 2. scRNA-seq reveals a number of potential trajectories of cellular changes in disease pathogenesis, differentiation characteristics, therapeutic targets etc. It is currently used by most scholars to study the pathogenesis, clinical detection and therapeutic effects of various kidney diseases. Some of the more common diseases including: lupus nephritis (LN), renal cell carcinoma (RCC), diabetic nephropathy (DN), acute kidney injury (AKI) and immunoglobulin A nephropathy (IgAN) were summarized.

**Table 2:** The studies on renal diseases using scRNA-seq

Diseases	Species	Cells or Samples	Cell Number	Device	Findings	Ref.
LN	human	skin/kidney	1584 single cell libraries	Fluidigm C1	scRNA-seq can be used in skin biopsies to detect biomarkers of kidney disease	Der et al. 2017
	human	skin/kidney	250 cells of each type		Heterogeneity and possible fibrotic pathways in LN	Der et al. 2019
	human	kidney/urine /blood	2881 cells	CEL-Seq2	Detailed view of the active 21 leukocyte subpopulations in the kidney of a patient with LN	Arazi et al. 2019
RCC	human/ mouse	primary renal cell carcinoma/metastatic renal cell carcinoma		Fluidigm C1	Potential applications of scRNA-seq for precision anti-cancer therapy	Kim et al. 2016
	human	primary renal cell carcinoma/metastatic renal cell carcinoma	118 cells		Heterogeneity of cancer cells in different subpopulations can activate different pathways and subdifferential markers in subpopulations are associated with intra-tumour heterogeneity, drug sensitivity and prognosis in renal cell carcinoma	Liu et al. 2021
	human	primary tumour cells/lymph node metastatic tumour cells/bone metastatic tumour cells	15,208 cells	10X GenomicsChromium	Differentiation process of tumour stem cells and genes associated with poor prognosis in collecting ductal renal cell carcinoma	Pan et al. 2020
	human	renal cell carcinoma benign adjacent renal tissue cells	20,500 cells	10X GenomicsChromium	Renal cell carcinoma benign adjacent renal tissue cells	Zhang et al. 2021
	human	kidney		10X GenomicsChromium	Revealing and comparing neutrophil subpopulations in the healthy kidney and tumour microenvironment facilitates the understanding of the heterogeneity and pathological significance of neutrophils in kidney disease	Meng et al. 2021
	human	kidney		10X GenomicsChromium	HIF-1α is mainly expressed in tumor-associated macrophages; HIF-2α and hypoxia-related factors are mainly expressed in tumor cells. HIF-1α can be used as a therapeutic target and disease progression marker in clear renal cell carcinoma	Cowman et al. 2020
	human	Blood/ tumour/ renal parenchymal samples	37,055 cells	10X GenomicsChromium	Different transcriptional states of tumour-infiltrating CD8^+^ T cells reveal certain immune cell subsets vulnerable to therapeutic intervention	Borcherding et al. 2021
DN	mouse	glomerular cells	644 cells	Fluidigm C1	Unravelling the dynamics of gene expression in the diabetic kidney and the differential response of individual cells to diabetic injury	Fu et al. 2019
	human	kidney cells/ immune cells	21,529 nuclei	10X GenomicsChromium	Cellular crosstalk in DN, the relationship between key genes for cellular communication and renal function	Wei et al. 2021
	human	renal cortical cells	23,980 nuclei	10X GenomicsChromium	Single-cell transcriptome map of early DN in humans	Wilson et al. 2019
AKI	human	Urine	30,076 cells	10X GenomicsChromium	Differences in cell composition and gene expression during AKI, several inflammatory immune cell populations and differential activation pathways	Cheung et al. 2022
	mouse	kidney	26,643 cells	10X GenomicsChromium	Gene expression changes during the repair of AKI, suggesting new therapeutic targets	Kirita et al. 2020
	mouse	kidney	54,730 cells	Droplet-based system	The first comprehensive renal cell type-specific transcriptional profile, potentially pathological epithelial interstitial crosstalk	Rudman-Melnick et al. 2020
	mouse	glomerular cells	75,000 cells	10X GenomicsChromium	Comprehensive high-resolution single-cell transcriptomic profiles generated, providing a resource for identifying novel disease-associated genes and pathways	Chung et al. 2020
	human/ mouse	Kidney cells/immune cells/epithelial cells		10X GenomicsChromium	Spatial transcriptome characterisation of AKI in a mouse model and demonstrates how the approach can be applied to human kidney tissue	Melo Ferreira et al. 2021
	human	kidney	23,367 cells	10X GenomicsChromium	Predicted potential pathways of renal injury through angiotensin-converting enzyme 2 (ACE2) in 2019 coronavirus disease	He et al. 2020
	mouse	kidney	8732 cells	10X GenomicsChromium	Ferroptosis-related genes were mainly expressed in tubular epithelial cells after I/R injury	Zhao et al. 2020
IgAN	human	kidney/ peripheral blood	2785 Kidney cells/835 peripheral blood mononuclear cells	10X GenomicsChromium	This study provided a promising prospect for disease treatment and suggested a role for CD8^+^ T-cell immune dysfunction in IgAN progression	Zheng et al. 2020
	mouse	kidney		Smart-seq2	The role of endothelial cells in immune cell recruitment, paracrine pathways within the glomerulus associated with inflammation promotion	Zambrano et al. 2022
Glomerulonephritis	human	kidney	14,932 cells	10X GenomicsChromium	Podocyte markers of glomerulonephritis, specific markers of IgA, membranous nephropathy and LN at the cellular level	Chen et al. 2021
Other	mouse	glomerular endothelial cells	40,000 cells	10X GenomicsChromium	High-resolution mapping of the renal endothelium, phenotypic heterogeneity of renal endothelial cells, responses and expression of endothelial cell involvement in hypertonic and dehydration responses	Dumas et al. 2020
	human	healthy kidney Cells/transplanted kidney Cells	4487 cells	InDrops	Heterogeneity of the immune response in mixed rejection reactions	Wu et al. 2018
	mouse	kidney	57,979 cells	Droplet-based system	Most genetic disorders of the kidney can be traced back to a single cell type. the relationship between the renal collecting duct system and diseases such as metabolic acidosis, chronic kidney disease and blood pressure	Park et al. 2018

### Lupus nephritis

Systemic lupus erythematosus (SLE) is a systemic autoimmune disease that usually affects all organs. About 50% of these patients will suffer from LN, in which severe cases can lead to kidney failure or even death. Abnormalities in the innate and adaptive immune response are one of the causes of LN. It is mainly characterised by the production of autoantibodies against nuclear and cellular antigens, which leads to the accumulation of immune complexes in the glomeruli. However, the detailed pathogenesis of the disease still remains unclear. Based on scRNA-seq of skin and kidney tissue from healthy individuals and patients with LN, Der et al. found active IFN signalling presented in major tissue constituent cell populations (renal tubular cells, keratinocytes, fibroblasts, endothelial cells, T cells and myeloid cells) in the kidneys and skin of patients with LN. Also, IFN responses were observed in skin keratin-forming cells in these patients that had not been damaged and exposed to sunlight (Der et al. 2017). This finding confirmed Blomberg’s finding that IFN-α producing cells (IPC) can be recruited not only to inflammatory sites in SLE, but can also be actively present in non-inflammatory sites (Blomberg et al. 2001). In addition, this phenomenon can expose levels of IFN-α or IFN-α produced in situ by resident, infiltrating cells in the systemic circulatory system. In this experiment, the authors tested skin tissue from SLE patients with LN, but lost skin tissue from lupus patients with healthy kidneys, so that the specificity of LN could not be known. Also, possibly due to the small number of samples in the experiment, no B cells were captured and only a small number of T cells were sequenced in this experiment compared to other experiments. In terms of heterogeneity in LN, Der et al. further collected kidney tissue from 21 patients with LN and healthy control subjects, revealed differences in the histological types of different LN through a large number of single cell sequencing data. They confirmed upregulation of type I IFN signalling genes in tubular cells not just infiltrating cells from patients with LN. In addition, they found significant upregulation of genes for extracellular matrix (ECM) proteins and ECM-interacting proteins by differential analysis of mean tubular cell profiles from each patient, suggesting that both related signalling pathways may be associated with renal tubulointerstitial fibrosis, which may represent a separate pathway of fibrosis (Der et al. 2019). Both studies found that type I IFN-response genes in tubular cells and keratinocytes from patients with LN were much more highly expressed than those of healthy control subjects, which demonstrated that single cell sequencing technology can be used to study kidney biopsies clinically obtained and suggested that skin biopsies could be used as a biomarker for kidney disease. Arazi et al. analysed kidneys, urine and blood from healthy individuals and patients with LN to reveal 21 leukocyte subpopulations were active in the disease and provided a detailed view of the immune cell population in the kidneys of patients. They found local activation of B cells correlated with an age-associated B-cell signature and progressive stages of monocyte differentiation within the kidney. The gene expression of urine immune cells was highly correlated with that of kidney immune cells, which would suggest that urine might serve as a surrogate for kidney biopsies (Arazi et al. 2019). These studies indicated that scRNA-seq can improve the understanding of immune cell infiltration in LN and can be applied to clinical treatment and diagnosis (Rao et al. 2020). scRNA-seq therefore provides a new tool to study the pathogenesis, clinical diagnosis and treatment of LN.

### Renal cell carcinoma

RCC is a malignant tumour arising from the epithelial cells of the renal tubules and is one of the most common tumours. According to the pathological subtypes, it can be divided into clear cell renal carcinoma (ccRCC), papillary renal cell carcinoma, chromophobe renal carcinoma and rare renal cell carcinoma (Udager and Mehra 2016). ccRCC is the most common of these, at around 70% or more (Saad et al. 2019).

Zhang et al. predicted cellular origin hypotheses for more than 10 renal cell carcinoma subtypes and found that overall survival was higher in patients with a higher proportion of endothelial cells in primary RCC, but there was no significant difference in metastatic RCC. In addition, they found that tumour epithelial cells could promote immune cell infiltration. These findings implied that RCC biology may be closely related to the tumour microenvironment (Zhang et al. 2021). To better characterise the tumour immune microenvironment in ccRCC, Borcherding et al. assessed tumour-specific transcriptional and clonal changes in the immune population at the single-cell level using three ccRCC tumour samples and immune cells from blood and renal parenchyma. They found significantly altered gene expression in tumour-infiltrating T cells and increased numbers of CD8^+^ T cells and macrophages in tumour-infiltrating immune cells compared to peripheral blood and normal renal parenchyma. In addition, they identified different transcriptional states of tumour-infiltrating CD8^+^ T cells and identified MKI67^+^ proliferative subpopulations as a potential factor in the development of ccRCC. These findings may allow for the discovery of certain immune cell subsets that can be used to treat ccRCC (Borcherding et al. 2021). Meng et al. investigated the heterogeneity of neutrophils in ccRCC by scRNA-seq and found that neutrophils in the kidneys of healthy individuals could be divided into two subpopulations, one involved in autoimmunity, which may serve as a therapeutic target for autoimmune kidney diseases, and the other associated with resistance to infectious diseases. In the kidneys of patients with ccRCC, neutrophils showed a higher degree of heterogeneity and were divided into six subpopulations. There was an overlap in the immunological/pathological profile of some subpopulations due to the upregulation of a large number of gene expressions. However, the first two populations are thought to be more strongly associated with autoimmune disease, while the latter four subpopulations may be involved in the development of cancer and may serve as therapeutic targets against cancer (Meng et al. 2021).

Approximately 30% of RCCs are metastatic kidney cancers, which appear in other areas, such as the brain, bones, liver, and lungs. There were different genomes and transcriptomes between primary RCC and metastatic renal cell carcinoma (mRCC) (Gupta et al. 2008). Tumour metastasis and heterogeneity are often the cause of drug resistance and death in patients with mRCC. In the treatment of mRCC, Kim et al. presented a pioneering study, in which they used scRNA-seq to explore the heterogeneity of mRCC and designed the optimal combination of targeted drugs through the activation of different drug pathways to derive a personalized treatment plan (Kim et al. 2016). Although this treatment option was not ultimately used clinically, it presented a new idea for the treatment of mRCC, and improved insight into cellular heterogeneity and more effective treatment of mRCC. Liu et al. used a combination of bulk sequencing and scRNA-seq to identify two major functional subgroups of mRCC: the epithelial-to-mesenchymal transition pathway-related cluster (EMT-RC) subpopulation and the vascular endothelial growth factor pathway-related cluster (VEGF-RC) subpopulation. They demonstrated transcriptomic heterogeneity of tumours and suggested that a combination of anti-VEGF therapy and anti-EMT therapy may be effective in patients with mRCC (Liu et al. 2021).

### Diabetic nephropathy

More than 700 million diabetic patients are expected by 2045, and more than 40% of them may develop DN and end-stage renal disease (ESKD), requiring dialysis or transplantation (Group 2020). However, the mechanism of DN is complex and not fully elucidated. With the development of scRNA-seq, Wei et al. downloaded single-nucleus transcriptomic(snRNA-seq) data from the Gene Expression Omnibus (https://www.ncbi.nlm.nih.gov/geo) dataset GSE131882. The snRNA-seq data from early DN patients and non-diabetic controls, which revealed the correlation between cell communication and renal function. In DN, the most obvious changes in glomerular and glomerular tubular cell crosstalk, cell-to-cell interactions via integrin pathways were increased, mesangial cells were stimulated and glomerulotubular communication was strongly enhanced. They found the correlation between glomerular FGF1 levels and glomerular filtration rate (GFR) levels, while glomerular NRP1, tubular COL4A1, and tubular NRP1 levels were negatively associated with GFR levels; suggesting that cell crosstalk-related mechanisms contributed to the development of DN. They also found that glomerular endothelial cell-expressed FLT1 and podocyte-expressed VEGFA and FGF1 are key molecules. FGF1 supplementation ameliorates DN through anti-inflammatory and antioxidative stress mechanisms, suggesting that FGF1 is a renoprotetctive factor and therapeutic target in DN (Wei et al. 2021; Group 2020). Wilson et al. compared 23,980 monocyte transcriptomes generated from three patients with early DN and three controls, and found that the thick ascending limb (TAL), late distal curved tubules, and principal cells all had gene expression features, including changes in the expression of Na + / K + -ATP enzyme (A. NKA), WNK lysine-deficient protein kinase 1 (WNK1), halocorticoid receptor and NEDD4L, which together promoted potassium secretion while reducing intercellular calcium and magnesium reabsorption. The reduction of A. NKA, inward rectifier potassium channel J subfamily members 16 (KCNJ16), and Na ^+^ -K ^+^ -2Cl-cotransporter (NKCC2) activity in TAL impaired transcellular sodium and potassium reuptake and reduced paracellular reuptake of calcium and magnesium, which was exacerbated by increased expression of the calcium-sensitive receptor (CaSR) and decreased expression of the CLDN16 that regulated tight junction permeability. These changes were accompanied by increased expression of serum-glucocorticoid-regulated kinase 1 (SGK1) in the collecting duct and decreased expression of ENaC, NEDD4L, an important regulator of potassium secretion. Furthermore, strong angiogenic signals were identified in near tubules, distal tubules and principal cells. Their study showed that the early response in DN increased the production of potassium and angiogenic signals (Wilson et al. 2019). These studies mentioned above once again highlight the ability of scRNA-seq to provide cell-specific information in kidney disease.

### Acute kidney injury

AKI is a group of syndromes characterized by a sudden decline in renal function, accompanied by significant mortality rates and increasing hospitalization rates. AKI can trigger or develop into chronic kidney disease (CKD), and eventually develops into ESKD. Exploring the molecular pathogenesis of the early AKI is critical for the development of therapeutic and diagnostic tools. Despite important advances in recent studies of AKI, the pathogenesis of AKI at the cellular and molecular levels remains incompletely unclear. Melo Ferreira et al. utilized single-nuclear and scRNA-seq data sets to map cell types back to spatial transcriptomic anchoring landmarks (spots) overlaid upon the human and murine kidney, and found that single-cell and spatial transcriptomic data sets are complementary and synergistic. The gene expression levels of individual cells in the healthy kidney and acutely injured kidney can map directly to tissue spatial locations. These tools will improve the ability of renal pathologists to interpret the mechanism and diagnose of AKI (Melo Ferreira et al. 2021). Chung et al. performed a scRNA-seq analysis using approximately 75,000 cells of purified glomeruli, providing a comprehensive transcriptional characterization of all cell types in the glomeruli, while also providing a comprehensive single-cell atlas. Meanwhile, the response of glomerular cells to four common types of kidney injury (immune, metabolic, toxic, and genetic injury) was observed. They generated a single-cell dataset focused on glomerular cells that can be used to generate precise tools to query specific glomerular cell types, as well as to identify genes involved in the pathogenesis of glomerular diseases (Chung et al. 2020). Results from four disease models provide new insights into the glomerular response to acute injury and its progression to CKD. Rudman-Melnick et al. performed scRNA-seq with the clinicall relevant unilateral ischemia-reperfusion model in mice at multiple AKI stages and found elevated expression of crucial injury response factors including kidney injury molecule-1 (Kim1), lipocalin 2 (Lcn2), and keratin 8 (Krt8)—and several novel genes (Ahnak, Sh3bgrl3, and Col18a1) not previously examined in kidney pathologies (Rudman-Melnick et al. 2020). AKI induced proximal tubule dedifferentiation with a marked nephrogenic signature and caused the formation of surprising mixed-identity cells (expressing markers of different renal cell types) in the injured renal tubules, which identified potential pathological epithelial stromal crosstalk. This work provides a rich resource for investigating the molecular genetics of AKI. Using single-nucleus RNA sequencing from the AKI mouse model to describe the cellular state during acute injury repair, Kiritaa et al. identified a distinct proinflammatory and profibrotic failed-repair proximal tubule cell (FR-PTC) state. Targeting this state reduced chronic inflammation and fibrosis after injury, suggesting that the FR-PTC state may represent a therapeutic target to improve repair (Kirita et al. 2020). Cheung et al. performed scRNA-seq on urinary sediment from hospitalized patients with COVID-19, assessed differences in cell composition and gene expression during COVID-19-associated AKI, and found that most of them were renal cells, urothelial cells, and immune cells. They also found several inflammatory immune cell populations and differentially activated pathways in COVID-19–associated AKI. The enrichment of reactive oxygen species (ROS), DNA damage, and apoptotic pathways in the proximal tubules, loop of Henle, and collecting duct were more apparent in COVID-19–associated AKI compared to the non–COVID-19 AKI. The transcripts of injury-related pathways were enriched in renal tubular cells from patients with COVID-19-related AKI compared to those without AKI, and pathways involved in type 1 interferon signalling, acute inflammatory response and leukocyte response were enriched in urothelial cells; pathways associated with apoptosis, DNA repair and cellular response to ROS and stress were enriched in proximal tubular cells. Furthermore, they established a dataset including both damaged and undamaged kidney cells, which provided preliminary evidence that SARS-CoV-2 is capable of directly infecting urothelial cells (Cheung et al. 2022). Transcriptional changes in single cell types in scRNA-seq provide unique insights into the pathogenesis of naive AKI and are providing possibilities to identify novel therapeutic strategies for AKI.

### IgA nephropathy

IgAN, a typical autoimmune disease, is the most common primary glomerulonephritis worldwide and represents the leading cause of kidney failure. Tang et al. presented a scRNA-seq analysis of human renal biopsies from IgAN. Their results showed for the first time that IgAN mesangial cells displayed increased expression of several novel genes including MALAT1, GADD45B, SOX4, and EDIL3, which were related to cell proliferation and matrix accumulation. Furthermore, the overexpressed genes in tubule cells from IgAN patients were mainly enriched in inflammatory pathways including TNF signaling, IL-17 signaling, and NOD-like receptor signaling (Tang et al. 2021). Zheng et al. performed scRNA-seq of the kidney and CD14^+^ peripheral blood mononuclear cells (PBMC) collected from IgAN patients and healthy donors to compare the molecular differences between normal and IgAN mesangial cells and identified JCHAIN was one of the most significantly upregulated genes in IgAN mesangial cells, which was available to identify and transport IgA and deposit IgA in the glomerular mesangial, leading to IgA nephropathy. High-level JCHIAN expression was characterized diffuse mesangial deposition of IgA, revealing a potential correlation with specific IgA deposition in the mesangium IgAN, which was proposed as a therapeutic target. In addition to JCHAIN, they uncovered the expression patterns of other genes in IgAN mesangial cells, involved in inflammatory signals and ECM accumulation, were specifically expressed in IgAN mesangial cells, and mesangial cells can serve as semiphagocytes to clear Ig types and the ECM. Genes expression profiles of IgAN may cause an imbalance between production and clearance, leading to progressive renal injury and glomerulosclerosis. Meanwhile, they further observed changes in T cells of healthy donors and IgAN patients and found that the levels of FCGR3A, GZMB, KLRD, FGFBP2, and GZMH, strongly related to T cell cytotoxicity, was markedly decreased in IgAN CD8^+^ T cells compared to normal CD8^+^ T cells. These results suggested the role of immune dysfunction of CD8 + T cells in IgAN progression (Zheng et al. 2020). All of these provide rich insights into the underlying mechanisms of IgA nephropathy.

## Conclusion

The advent of scRNA-seq has led to a more comprehensive understanding of the pathogenesis of kidney disease. The rapid development of this technology has led to a wide range of applications in the detection, evaluation and treatment of kidney disease. By reviewing the relevant literature, we described the applicability and advancement of scRNA-seq in the study of the pathogenesis of kidney disease. By summarising the applications of scRNA-seq in this field, we found that scRNA-seq technology can comprehensively analyse cell types in different renal diseases, improve the ability to characterise cells in heterogeneous organs such as the kidney, deepen the understanding of the heterogeneity of tissue samples, and provide new ideas for the discovery of therapeutic targets in renal diseases.

Nevertheless, there are several problems with this technology at this stage: firstly, scRNA-seq has not yet been widely translated into clinical practice, and there is a need to improve its diagnostic and prognostic accuracy. It is difficult to measure mRNA expression in transcriptomes superimposed on the same tissue section only by a single cell sequencing technique, and integration with spatial transcriptomics is required for a comprehensive measurement. Secondly, in the case where obtaining human kidney biopsy is relatively difficult and the small specimen size, the requirement for obtaining valid and accurate data for scRNA-seq is relatively high. Thirdly, this technology does not provide direct measurements of microRNA, proteomic or metabolic data, although some insights can be inferred. Therefore, scRNA-seq needs to be used in conjunction with other omics to explore the complex etiology of nephrology.

## Electronic supplementary material

Below is the link to the electronic supplementary material.

Supplementary Material 1

## Data Availability

Not applicable.

## Abbreviations

scRNA-seq: single-cell RNA sequencing
UMI: unique molecular identifiers
IFCs: Integrated Fluidic Circuits
NGS: next-generation sequencing
ddPCR: droplet digital PCR
IFN-α: interferon alpha
IPC: IFN-α producing cells
LN: lupus nephritis
SLE: systemic lupus erythematosus
RCC: renal cell carcinoma
ccRCC: clear cell renal carcinoma
mRCC: metastatic renal cell carcinoma
EMT-RC: epithelial-to-mesenchymal transition pathway-related cluster
VEGF-RC: vascular endothelial growth factor pathway-related cluster
DN: diabetic nephropathy
ESKD: end-stage renal disease
snRNA-seq: single-nucleus transcriptomic
GFR: glomerular filtration rate
A. NKA: Na + / K + -ATP enzyme
TAL: the thick ascending limb
WNK1: WNK lysine-deficient protein kinase 1
NKCC2: Na + -K + -2Cl-cotransporter
CaSR: calcium-sensitive receptor
SGK1: serum-glucocorticoid-regulated kinase 1
AKI: acute kidney injury
CKD: chronic kidney disease
Kim1: kidney injury molecule-1
Lcn2: lipocalin 2
Krt8: keratin 8
FR-PTC: failed-repair proximal tubule cell
ROS: reactive oxygen species
IgAN: Immunoglobulin A nephropathy
PBMC: peripheral blood mononuclear cells
ECM: extracellular matrix
