# Supplementary material for: Single-cell RNA sequencing for the study of kidney disease
Source: Mol Med. 2023 Jul 3;29:85. doi: 10.1186/s10020-023-00693-8 (PMC10318824; doi:10.1186/s10020-023-00693-8)
Supplement: Supplementary file 1 — Supplementary Material 1 [file 10020_2023_693_MOESM1_ESM.docx]

Reviewer #1: Zhu et al. reviewed the use of scRNA-seq in the area of renal physiology and pathophysiology. The authors summarize the current platforms utilized in the field as well as examples of how this technique has been utilized in many renal related pathologies.

**Answer:** Thank you for your comments.
1) The overall tone of the review seems very casual or conversational. For example in the introduction, "In the early studies, kidney cells were studied using microscopic or fluorescence-activated cell sorting, which is not good at exploring the pathogenesis of kidney disease." It sounds very informal. Can the authors please edit these types of sentences to make the review more formal?

**Answer:** We have revised or deleted these informal descriptions. Line 4 from the bottom page 1 "In the early studies, kidney cells were studied using microscopic or fluorescence- activated cell sorting, which is not good at exploring the pathogenesis of kidney disease." and Line 6 page 2 “In fact, single-cell level researches on kidney diseases had been lagging behind the field of immunology, etc.” have been deleted. Line 3 page 1 "With the development of science and technology, more and more techniques are being used in the study of kidney diseases." has been changed to "In recent years the application of single-cell RNA sequencing (scRNA-seq) in nephrology has developed rapidly." Line 10 page 14 “DN, one of the major complications of diabetes mellitus, is characterized by both glomerular and tubulointerstitial injury.” and Line 13 page 14 “The main causes of DN include the poor lifestyle, the prevalence of type 2 diabetes, and the increased incidence of type 1 diabetes,” have been deleted.
2) Perhaps a summary table for the different types of equipment that are utilized to perform scRNA-seq analysis would be helpful for the reader.

**Answer:** We added "We summarized some of the most commonly used scRNA-seq platforms or methods, as shown in Table 1, providing a guide that could help nephrologists to choice scRNA-seq methods suitable for their studies." to line 2 page 3.

Table 1 Principles and characteristics of different single cell sequencing

| Methods | 10X Genomics Chromium System | Fluidigm C1 System | Illumina /BioRad |
| --- | --- | --- | --- |
| Transcript coverage | 5’ /3’ end-counting | Full-length | 100bp reads |
| Unique molecular identifiers (UMI) | Yes | No | Yes |
| Single cell isolation | Droplet-based microfluidic system | Microfluidic-based system | Fluorescence-activated cell sorting |
| Cell size limitations | Independent of cell size | Homogenous size of 5-10,10-17,or 17-25 μM | Independent of cell size |
| Cell number required | ≥20,000 | ≥10,000 | Hundreds~thousands |
| Throughput | High | Low | High |
| Precision | High | Medium | High |
| Cost | Low | High | Medium |
| Operation | Easy | Difficult | Easy |
| Run time (approximately 5000 single cells) | ~2-3 days | ~3 weeks | ~ 2 days |

3) There are a few places throughout the manuscript were the wrong word was chosen. For example, in the abstract it should be "we summarized the technical platform…". Please carefully proofread for manuscript.

Answer: We have corrected these errors. We have changed summaries to summarized (line 5 page 1); are to were (line 6 from the bottom page 6; line 1 from the bottom page 6; line 12 page 12; line 9 from the bottom page 13; line 5 from the bottom page 14); is to was (line 15 page 12; line 4 from the bottom page 14); show to showed (line 9 from the bottom page 13); reduces to reduced (line 6 from the bottom page 16);provides to provided (line 7 page 17).

Reviewer #2:
The review by Zhu et al entitled 'single-cell RNA sequencing for the study of kidney disease' summarizes both the currently utilized techniques in single cell RNAseq along with associated strengths and weaknesses as well as summarizing the findings of a number of such approaches in various kidney diseases to date.
Strengths: Overall the paper is generally well written and is likely to be of interest to scientists new to the technique.

The study does a good job describing differences between RNA seq approaches and the associated limitation of each approach.

**Answer**: Thank you for your comments
Weaknesses:
Some of the writing, particularly with relevance to kidney biology is too diffuse. For example Line 1 page 2 'reduce the limitations of different cell types on the pathogenesis of kidney disease' - the meaning is unclear.

**Answer:** We changed Line 8 page 2 “reduce the limitations of different cell types on the pathogenesis of kidney disease” to “Park et al. performed unbiased scRNA-seq of 57,979 kidney cells from healthy mice and successfully found that each cell type appeared to be important for the pathogenesis of kidney disease.”

Line 5 page 11 “It has been found that intrarenal expression of interferon alpha (IFN-α) promotes the production of antigen-presenting cells, CD4^+^ helper T cells, CD8^+^ memory T cells and the differentiation of B cells, ultimately leading to the formation of autoantibodies and immune complexes. IFN-α has also been shown to be associated with SLE, and IFN signalling is associated with the severity of renal disease(Parikh et al. 2020; Baechler et al. 2003).” These descriptions have been deleted.

Line 10 page 14 “DN, one of the major complications of diabetes mellitus, is characterized by both glomerular and tubulointerstitial injury.” Line 12 page 14 “The main causes of DN include the poor lifestyle, the prevalence of type 2 diabetes, and the increased incidence of type 1 diabetes,” These descriptions have been deleted.
Intro
Line 30 - you mean maintaining the 'internal' environment

**Answer**: We have added “internal” to this sentence. (Line 11 page 1)
Line 42 ' the complexity of kidney cells hinders the understanding of kidney disease' - You mean the heterogeneity of cell types in the kidney?

**Answer:** We have changed “complexity of kidney cells” to “heterogeneity of cell types in the kidney” (Line 5 from the bottom page 1); “Its cellular complexity” to “in which, the heterogeneity of cell types” (Line 2 page 1).
Line 44 'which is not good at exploring the pathogenesis of kidney disease' - seems like an overstatement to suggest microscopy of FACS are 'not useful'

**Answer:** We have deleted this sentence.(Line 4 from the bottom page 1)
Line 47 - with the advancement of science and technology..... sentence is too long and meaning hard to understand

**Answer**: We have changed line 3 from the bottom page 1 “With the advancement of science and technology, gene sequencing has become popular and is being used in various fields. Single-cell RNA sequencing (scRNA-seq) differs from other gene sequencing techniques in that it can determine the expression level of each gene in a single cell, which addresses the loss of cellular heterogeneity generated by the transcriptional of cell populations and can be of great help in identifying new cells, redefining known cells, and understanding cell differentiation.” to “Traditional RNA sequencing only evaluates the average gene expression of all different kinds of cells in the tissue. The rapid development of single-cell RNA sequencing (scRNA-seq) allows investigators to monitor global gene expression in individual cells, which can be of great help in identifying new cells, redefining known cells, and understanding cell differentiation.”
